# Cancer in Korean patients with end-stage renal disease: A 7-year follow-up

**DOI:** 10.1371/journal.pone.0178649

**Published:** 2017-07-10

**Authors:** Kyung Don Yoo, Jung Pyo Lee, Su Mi Lee, Jae Yoon Park, Hajeong Lee, Dong Ki Kim, Shin-Wook Kang, Chul Woo Yang, Yong-Lim Kim, Chun Soo Lim, Kwon Wook Joo, Yon Su Kim

**Affiliations:** 1 Department of Internal Medicine, Dongguk University College of Medicine, Gyeongju, Korea; 2 Department of Internal Medicine, Seoul National University Boramae Medical Center, Seoul, Korea; 3 Department of Internal Medicine, Dong-A University College of Medicine, Busan, Korea; 4 Department of Internal Medicine, Seoul National University College of Medicine, Seoul National University Hospital, Seoul, Korea; 5 Department of Internal Medicine, Yonsei University College of Medicine, Seoul, Korea; 6 Department of Internal Medicine, Catholic University of Korea College of Medicine, Seoul, Korea; 7 Department of Internal Medicine, Kyungpook National University Hospital, Daegu, Korea; Hospital Universitario de la Princesa, SPAIN

## Abstract

**Background:**

The effectiveness of dialysis on the incidence of cancer in patients with end-stage renal disease (ESRD) remains to be clarified. In this study, we evaluated the incidence rate and type of cancer among patients with ESRD, compared to the general population, through a prospective 7-year follow-up. We also calculated the cumulative incidence rate of cancer associated with ESRD, with stratification to control for the competing risk of death.

**Methods:**

This prospective observational cohort study was conducted using data from a nationwide study on patients with ESRD in Korea. A total of 5,235 patients, ≥18 years old, with ESRD were identified from the national registry as being treated by dialysis between August 2008 and December 2014. The standardized incidence ratio (SIR) and cumulative incidence rate of specific cancers were evaluated and compared to the general population.

**Results:**

A total of 5,235 participants were included. During the 7 year observation period, 116 (2.2%) participants had been diagnosed as cancer. The SIR of overall cancer was 0.94 [95% confidence interval (CI), 0.72–1.19] and was comparable to the rate for the general population. Although the digestive organs were the most frequent site of a primary site cancer (N = 39, 33.6%), the SIR was highest for urinary tract cancer [4.7, 95% CI, 2.42–8.19]. The five year standardized cumulative incidence of cancer was higher for females than for males, and for non-diabetic compared to diabetic causes of ESRD. We estimated that the five year standardized cumulative incidence was highest [8.4, 95% CI, 5.07–13.75] in patients with ESRD, caused by glomerulonephritis.

**Conclusion:**

A screening program should be necessary for urinary tract cancer in Korean patients with ESRD. Cancer screening programs for patients with ESRD in Korea should be emphasized on female patients and patients with non-diabetic ESRD.

## Introduction

The incidence of malignant tumors is increasing worldwide. Specifically in patients with end-stage renal disease (ESRD), cancer is the third leading cause of death, after cardiovascular disease and infection [1,2]. Several studies have reported a higher incidence rate of cancer among patient with ESRD than among the general population [3–8]. Although the exact mechanism underlying this increased risk of ESRD-related cancer has yet to be clarified, several hypotheses have been put forth regarding a possible effects of dialysis-related factors [3–6,9], including: uremia-induced immune dysfunction [9,10]; altered DNA repair and methylation [9,11]; and elevated serum levels of the specific carcinogen [9,12]. In Asian patients with ESRD, abuse of analgesics and Chinese herbs containing aristolochic acid, which is used to treat kidney disease, could increase the risk of genitourinary cancer [6–9].

Although current studies have estimated the rates of malignancy of the urinary system, including renal cell carcinoma [3–8] and bladder carcinoma [3–6,8], to be higher among patients with ESRD, compared to the general population, these estimations are confounded by methodological error in calculating the incidence ratio between-groups [4–6]. Moreover, use of a cancer diagnosis without pathological confirmation may introduce significant bias in the interpretation of findings [3]. The high mortality rate specifically associated with ESRD is another important factor to consider when calculating the risk of cancer in this clinical population [13]. In Korea, an accurate rate of the incidence and prevalence of cancer in patients with ESRD has not been conclusively established, with data, to date, having been based only on retrospective studies [14]. Consequently, diagnostic criteria for cancer screening among patients with ESRD have not been established in Korea. Therefore, the aim of our study was to evaluate the incidence rate and type of cancer among patients with ESRD, compared to the general population, over a 7-year follow-up period. We also calculated the cumulative incidence rate, with stratification to control for the competing risk of death associated with ESRD. A secondary aim was to define a possible diagnostic screening protocol for malignant disease for patients with ESRD in Korea.

## Materials and methods

### Data source and study participants

This study was conducted as one of the components of a large, nationwide, prospective cohort study of patients with ESRD led by the Clinical Research Center for End Stage Renal Disease (CRC for ESRD) in Korea. Thirty-one hospitals and clinics in Korea participated in the CRC for ESRD study, with 2,207 participants with a new cancer (incidence patients) and 3,028 participants with an identified cancer (prevalence patients), ≥18 years of age and receiving dialysis between August 2008 and December 2014, enrolled (Table 1). Our methods to identify patients on dialysis for the CRC for ESRD study (NCT00931970) has previously been described [15]. All study participants provided written informed consent for use their clinical data for research and publication. Our study was approved by the institutional review board (IRB) at each center. Seoul National University Hospital Institutional Review Board approved with IRB number H-0905-047-281. All clinical investigations were conducted in accordance with the guidelines of the 2008 Declaration of Helsinki.

**Table 1:** Between-group comparison of baseline characteristics based on cancer incidence.

Variables*		ESRD without cancer (N = 5,119)	ESRD with cancer (N = 116)	*P*^†^
Age (years)		56.2 ± 13.5	59.6 ± 12.1	*0*.*006*
Sex (male)		3004 (58.7%)	66 (63.5%)	0.327
Primary renal disease				0.131
	Diabetes	2226 (43.5%)	39 (37.5%)	
	Hypertension	938 (18.3%)	21 (20.2%)	
	Glomerulonephritis	704 (13.8%)	21 (20.2%)	
	Cystic kidney disease	139 (2.7%)	2 (1.9%)	
	Unknown	272 (5.3%)	1(1.0%)	
	Others	840 (16.4%)	20 (19.2%)	
Preexisting history of CVD		1498 (29.3%)	31 (29.8%)	0.904
History of diabetes		2485 (48.5%)	45 (43.3%)	0.287
Proportion of prevalent dialysis		2953 (57.7%)	75 (72.1%)	*0*.*003*
Dialysis modality (HD, N, %)		3314 (65.3%)	64 (61.5%)	0.424
Dialysis duration (month)		56.3 ± 52.0	73.4 ± 58.1	*0*.*004*
Current smoking history (%)		492 (9.6%)	8 (7.7%)	0.510
BMI (kg/m^2^)		22.8 ± 3.3	23.3 ± 3.6	0.118
Modified CCI		5.06 ± 2.25	5.52 ± 2.73	*0*.*040*
Antihypertensive medications (%)				
RAAS blockade		2274 (44.4%)	46 (44.2%)	0.969
Calcium channel blockers		2859 (55.9%)	55 (52.9%)	0.547
Beta-blockers		2420 (47.3%)	44 (42.3%)	0.315
Diuretics		2287 (44.7%)	46 (44.2%)	0.928
alpha-Blockers		653 (12.8%)	16 (15.4%)	0.427
Hemoglobin (g/dL)		9.9 ± 3.3	10.1 ± 1.9	0.667
Phosphorus (mg/dL)		5.2 ± 2.0	4.8 ± 1.5	0.103
Albumin (g/dL)		3.5 ± 0.6	3.4 ± 0.7	0.668
Cholesterol (mg/dL)		160 ± 43	159 ± 40	0.922
Creatinine (mg/dL)		8.56 ± 4.44	8.24 ± 4.36	0.518
Dialysis-related indexes				
Hemodialysis patients				
Single-pool Kt/V		1.44 ± 0.43	1.37 ± 0.29	0.248
Weekly Kt/V		4.16 ± 1.36	3.97 ± 1.04	0.295
Urea reduction rate (%)		68.82 ± 10.05	68.29 ± 8.96	0.708
UF/session (kg)		1.87 ± 1.59	1.90 ± 1.33	0.891
Weekly UF (kg)		5.47 ± 4.79	5.66 ± 4.09	0.750
Peritoneal dialysis patients				
Weekly Kt/V		2.55 ± 1.86	2.89 ± 1.33	0.569
Drain volume/day (ml)		7,816 ± 2,510	7,959 ± 2,257	0.777
Time to follow-up duration (months)		26.3 ± 18.9	38.3 ± 17.8	*<0*.*001*
Time to discovery of cancer from dialysis initiation (months)			60.2 ± 51.3	

CVD, cardiovascular disease; SD, standard deviation; ESRD, end-stage renal disease; MCCI, modified Charlson comorbidity index; RAAS blockade, Renin-angiotensin-aldosterone system Blockade; UF, ultrafiltration.

*Values are presented as n (%) for categorical variables, mean ± SD for continuous variables

†p value was obtained from bivariate analysis.

### Clinical parameters

Our methods for extracting medical history records from the overall CRC for ESRD cohort has previously been described (http://webdb.crc-esrd.or.kr) [15]. For our analysis, the following clinical parameters were extracted from the medical records at the time of enrollment into the CRC for ESRD study: history of cardiovascular disease, diabetes, and smoking; anti-hypertensive medication history; and dialysis-associated information, including the primary cause of ESRD, dialysis modality and dialysis duration. Dialysis duration was calculated as the time (months) between the date of initiation of dialysis and the date at the end follow-up. The following baseline laboratory findings, measured at the time of enrollment in the CRC for ESRD study, were also extracted for analysis: serum level of hemoglobin, calcium, phosphorus, protein, albumin, and cholesterol (Table 1).

### Outcome measurements

The incidence (and prevalence) of cancer was evaluated by conducting in-person interviews with the 5,235 participants forming the prospective cohort of the CRS for ESRD study. An event of cancer development was recorded by the date of diagnosis, primary site, staging at the time of diagnosis, and type of oncological treatment. The primary site of cancer was classified into one of the following nine sectors: unknown, digestive, urinary tract, respiratory, reproductive, head and neck, hematologic, endocrine, and other organ. Further subclassification of primary tumor location by site is shown in Table 2. Patients with a diagnosis of cancer at the time of enrollment were excluded from the analysis. Regions of metastasis were recorded; however, metastasis was recorded as a comorbid disease. Occurrence of a second site of cancer was reported a ‘new’ cancer incidence. Cancer staging at the time of diagnosis was classified as follows: unknown, in situ, invasive, regional lymph nodes, and distant metastasis. Oncological treatment was classified as follows: none, unknown, local excision, wide excision and graft, site excision and node dissection, radiotherapy, chemotherapy, excision and radiotherapy, excision and chemotherapy, and other.

**Table 2:** Mean interval to cancer identification and primary site of malignancy among ESRD patients.

	Primary site N (%)	Time to follow-up duration (months)	Time to identification of cancer from dialysis initiation (months)	Cancer location	N (%)
Digestive	39 (33.6)	37.8 ± 16.8	58.6 ± 48.7	Stomach	13 (11.2)
				Colorectal	16 (13.8)
				Liver	6 (5.2)
				Pancreatobiliary	2 (1.7)
				Other GI tract	2 (1.7)
Urinary tract	19 (16.4)	39.3 ± 17.6	52.0 ± 39.5	Kidney	12 (10.3)
				Bladder	5 (4.3)
				Others	2 (1.7)
Respiratory	15 (12.9)	36.4 ± 20.7	37.4 ± 35.5	Lung	14 (12.1)
				Larynx	1 (0.9)
Reproductive	9 (7.8)	30.2 ± 23.0	68.1 ± 46.9	Uterine	2 (1.7)
				Prostate	6 (5.2)
				Others	1 (0.9)
Head & Neck	3 (2.6)	40.3 ± 29.9	57.6 ± 19.4	Tongue	1 (0.9)
				Others	2 (1.7)
Hematologic	7 (6.0)	38.9 ± 10.1	37.2 ± 25.0	Multiple myeloma	1 (0.9)
				Lymphoma	3 (2.6)
				Others	3 (2.6)
Endocrine	12 (10.3)	43.5 ± 10.8	110.1 ± 87.7	Thyroid	11 (9.5)
				Others	1 (0.9)
Other organ	12 (10.3)	41.7 ± 21.4	59.0 ± 28.6	Breast	9 (7.8)
				Skin	3 (2.6)

### Statistical analysis

Differences in continuous variables between dialysis patients *with* and *without* a cancer event were evaluated using unpaired *t*-test: age, body mass index, dialysis duration, Charlson comorbidity index, and serum blood laboratory levels. Between-group differences in categorical variables, comorbidities and medication history, were compared using a chi-squared test. All statistical analyses were performed using SPSS (IBM Corp. IBM SPSS Statistics for Windows, Version 19.0. Armonk, NY, USA), SAS version 9.3 (SAS Institute, Cary, NC, USA) and R statistical language (Version R 3.0.2, The Comprehensive R Archive Network: http://cran.r-project.org).

Standardized incidence ratios (SIR) were used to compare the cancer incidence rate between patients with ESRD and the general population, where the incidence rate of cancer among the general population was calculated using the National Cancer Registry statistics for the years 2009–2012 [16]. The SIR was calculated using an indirect standardization method, with the expected number of events calculated using a person-year method [17, 18]. The 95% confidence interval (CI) was calculated based on the expectation that the number of observed events followed a Poisson distribution [18]. To calculate the number of expected events, we multiplied the age- and sex-specific incidence rates obtained from National Cancer Registry statistics, for the years 2009–2012, by the number of person-years at risk for cancer in each age and sex categories in our study population.

Considering ESRD-related death as a competing risk, the crude cumulative incidence rate of cancer was estimated using Gray’s method [19–21]. Taking into account baseline characteristics, we applied the inverse probability of treatment weight (IPTW), using the marginal proportion of baseline characteristics exposure, in our calculation of standardized cumulative incidence rate estimates [22]. When the IPTW was set to the inverse of the propensity score by weight, we used the stabilized weight to minimize the effect of weight variation on estimates of the standardized cumulative incidence rate [23]. The following factors, measured at baseline using inverse-probability weights, were accounted for in the calculated standardized cumulative incidence rates: age at the initiation of dialysis, sex, primary cause of ESRD, dialysis duration, dialysis modality, and diabetes. At final, we reviewed data to minimize the possibility of bias from extreme weight size. S1 Table shows the result of estimated weight size, and we confirmed that there were no extreme values for requirement of additional adjustment. For analysis, age was stratified by interquartile range as follows: 25^th^ percentile, ~47 years of age; 50^th^ percentile, >47 years to 57 years; 75^th^ percentile, >57 years to 67years; and 100^th^ percentile, >67years. During our statistical analysis, a total of 656 patients were excluded because of missing data needed to calculate propensity scores. Therefore, our final analysis was based on the data of 4,579 patients.

## Results

### Baseline characteristics

The baseline characteristics of patients with a recorded cancer event are summarized in Table 1, including a comparison of baseline characteristics to patients *without* a recorded cancer event. Of the 5,235 patients forming our study group, 116 (2.2%) participants were diagnosed with a malignancy. The average duration from the initiation of dialysis to the cancer identification was 60.2 months. Within our study group, patients with cancer were older and had a higher comorbidity index. Moreover, the duration of dialysis was longer in patients *with* cancer than in those *without* cancer (73.4 *versus* 56.3 months, respectively, p = 0.01). The observation period, from the point of enrollment into the study, was significantly longer for patients *with* cancer than those *without* cancer (38.3 *versus* 26.3 months, respectively, P <0.001). There were, however, no differences in the distribution of sex, dialysis modality, causes of ESRD, and laboratory findings among patients *with* and *without* cancer.

### The primary site of cancer and average interval to identification

The primary site of malignancy is reported in Table 2. The digestive organs were the primary site of malignancy (33.6%, 39/116 cases), followed by the urinary tract (16.4%, 19/116 cases) and the respiratory tract (12.9%, 15/116 cases). The most common cancer was colorectal cancer, followed by lung cancer and kidney cancer. The time lapse from enrollment into the study to cancer identification, at the primary site, was 38.3 ± 17.8 months (Table 1). When considering all cancer events, the average time from dialysis initiation to cancer diagnosis was 60.2 months.

### Standard incidence ratios of cancer by primary site and age distribution

The standardized incidence ratio (SIR) of overall cancer was 0.94 [95% CI, 0.72–1.19] (Table 3). The incidence rate of cancer among our study group of patients with ESRD was not significantly different compared with that of the general population. Although we identified the digestive organs as the most frequent site of cancer (N = 19), the SIR for this cancer did not differ from that for the general population. When the SIR was calculated according to cancer type, the incidence rate of urinary tract cancer was 4.7-fold higher among patients with ESRD than among the general population (Table 3). However, the incidence rates of cancer in patients with ESRD, overall, were not different compared with those expected in the general population across all age groups (Table 4).

**Table 3:** Standard incidence ratios of cancer among ESRD patients by primary site of the cancer, diagnosed between 2009 and 2012.

Site	Person year	Predicted	Expected	SIR^a)^[95% CI]^b)^
All	7835.07	66	70.2	0.939 [0.727, 1.195]
Digestive Organ	7872.40	19	31.2	0.607 [0.365,0.948]
Urinary tract	7877.21	12	2.5	4.691 [2.424,8.195]
Respiratory tract	7880.50	8	8.7	0.916 [0.395,1.805]
Reproductive organ	7881.17	6	5.8	1.023 [0.375,2.227]
Head & Neck	-	0	-	-
Hematologic	7879.51	5	2.5	1.997 [0.648,4.662]
Endocrine organ	7878.04	8	9.8	0.809 [0.349,1.594]
Other organ	7882.68	8	4.8	1.650 [0.712,3.252]

a) Age, sex adjusted overall SIR

b) Exact 95% Confidence Interval method

**Table 4:** Standard incidence ratios of cancer among ESRD patients by age, diagnosed between 2009 and 2012.

Site	Person year	Predicted	Expected	SIR^a)^[95% CI]^b)^
Under 40 years	883.05	1	1.4	0.668[0.016,3.724]
40~49	1345.85	10	5.4	1.838[0.881,3.380]
50~59	2166.53	15	15.3	0.978[0.547,1.614]
60~69	2002.85	24	22.9	1.045[0.669,1.555]
Over 70 years	1436.8	16	25.1	0.636[0.364,1.034]

a) Sex adjusted overall SIR

b) Exact 95% Confidence Interval method

### Detail information of urinary tract malignancy in patients with ESRD

In part of the SIR results, we clarified that the incidence rate of urinary tract cancer among our study participants was significantly higher compared with that of the general population (Table 3). Further details regarding the information associated with the prevalence of cancer types (renal and urological), cancer stage at the diagnosis, treatment options, and the causes of ESRD were presented at Table 5. As we expected, clear-cell type renal cell carcinoma (RCC) was the most common in kidney cancer (66.6%), and urothelial carcinoma was the most common in bladder cancer (75.0%). The RCC was mostly founded in a non-metastatic state at the time of cancer diagnosis, therefore surgical treatment option was mainly applied (75.0%). On the other hand, bladder cancer was frequently founded in metastatic state at the time of cancer diagnosis (75.0%). In addition, we presented detailed information associated with the stage at diagnosis and treatment options in the other type of cancers (S2 Table).

**Table 5:** Detail information of newly developed urinary tract cancer in patients with ESRD.

	Primary Cause of ESRD (%)		Cancer type (%)		Cancer Stage at diagnosis (%)		Treatment Type of Cancer (%)	
Kidney (N = 12)	DM	1 (8.3)	RCC,clear cell type	8 (66.6)	Unknown	2 (16.7)	None	1 (8.3)
	HTN	3 (25.0)					Unknown	0 (0.0)
	GN	3 (25.0)			In situ	6 (50.0)	OP only	9 (75.0)
	Cystic kidney disease	0 (0.0)	Unknown	4 (33.3)			RT or CT only	0 (0.0)
	Unknown	3 (25.0)			Metastasis	4 (33.3)	OP + RT or CT	0 (0.0)
	Others	2 (16.6)					Others	2 (16.7)
Bladder (N = 5)	DM	1 (20.0)	Urothelial carcinoma	4 (75.0)	Unknown	1 (50.0)	None	0 (0.0)
	HTN	2 (40.0)					Unknown	0 (0.0)
	GN	0 (0.0)			In situ	0 (0.0)	OP only	3 (60.0)
	Cystic kidney disease	0 (0.0)	Unknown	1 (25.0)			RT or CT only	0 (0.0)
	Unknown	0 (0.0)			Metastasis	4 (80.0)	OP + RT or CT	0 (0.0)
	Others	2 (40.0)					Others	2 (40.0)
Others (N = 2)	DM	0 (0.0)	Renal pelvis, Infiltrating urothelial carcinoma	1 (50.0)	Unknown	1 (50.0)	None	0 (0.0)
	HTN	0 (0.0)					Unknown	1 (50.0)
	GN	0 (0.0)			In situ	0 (0.0)	OP only	1 (50.0)
	Cystic kidney disease	0 (0.0)	Unknown	1 (50.0)			RT or CT only	0 (0.0)
	Unknown	1 (50.0)			Metastasis	1 (50.0)	OP + RT or CT	0 (0.0)
	Others	1 (50.0)					Others	0 (0.0)

ESRD, end-stage renal disease;DM, diabetes mellitus, HTN, hypertension; GN, Glomerulonephritis RCC, renal cell carcinoma; OP, operation; RT, radiotherapy; CT, chemotherapy

### The five-year standardized cumulative incidence ratio

**Fig 1:**
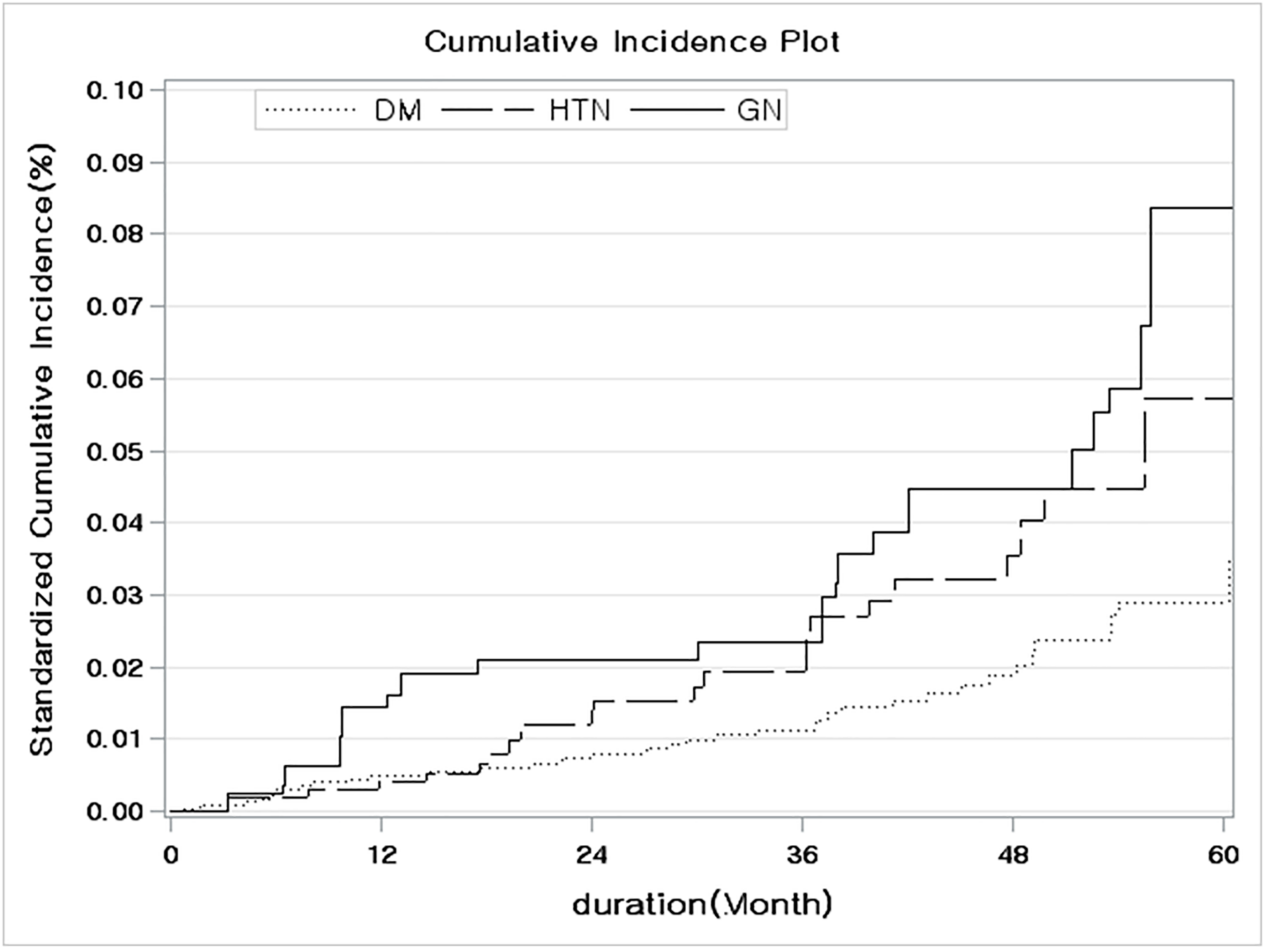
Comparison of the standardized cumulative incidence ratio controlling for the competing risk of death from any other cause stratified by primary cause of end-stage renal disease.

**Fig 2:**
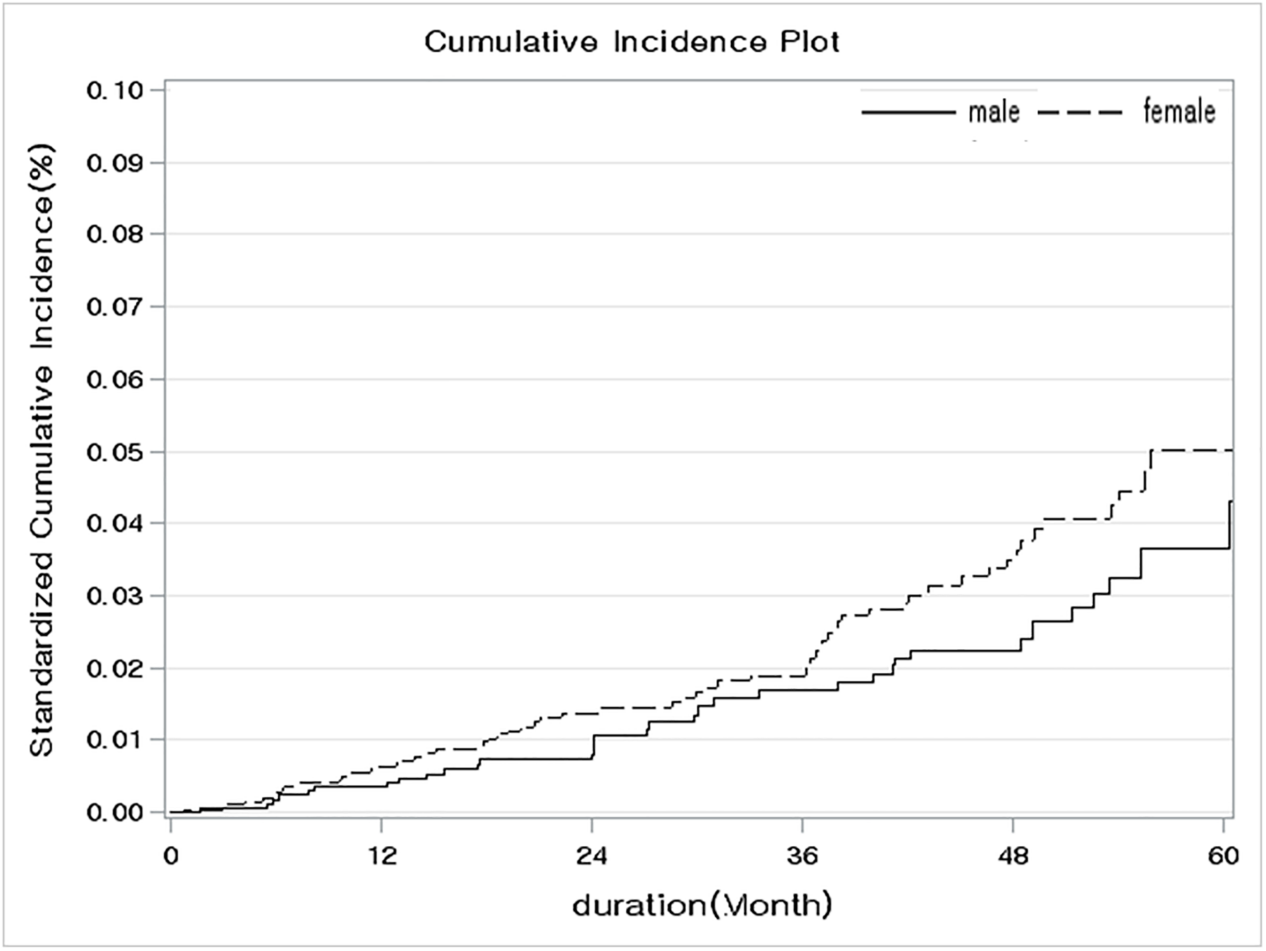
Comparison of the standardized cumulative incidence ratio controlling for the competing risk of death from any other cause stratified by sex.

**Fig 3:**
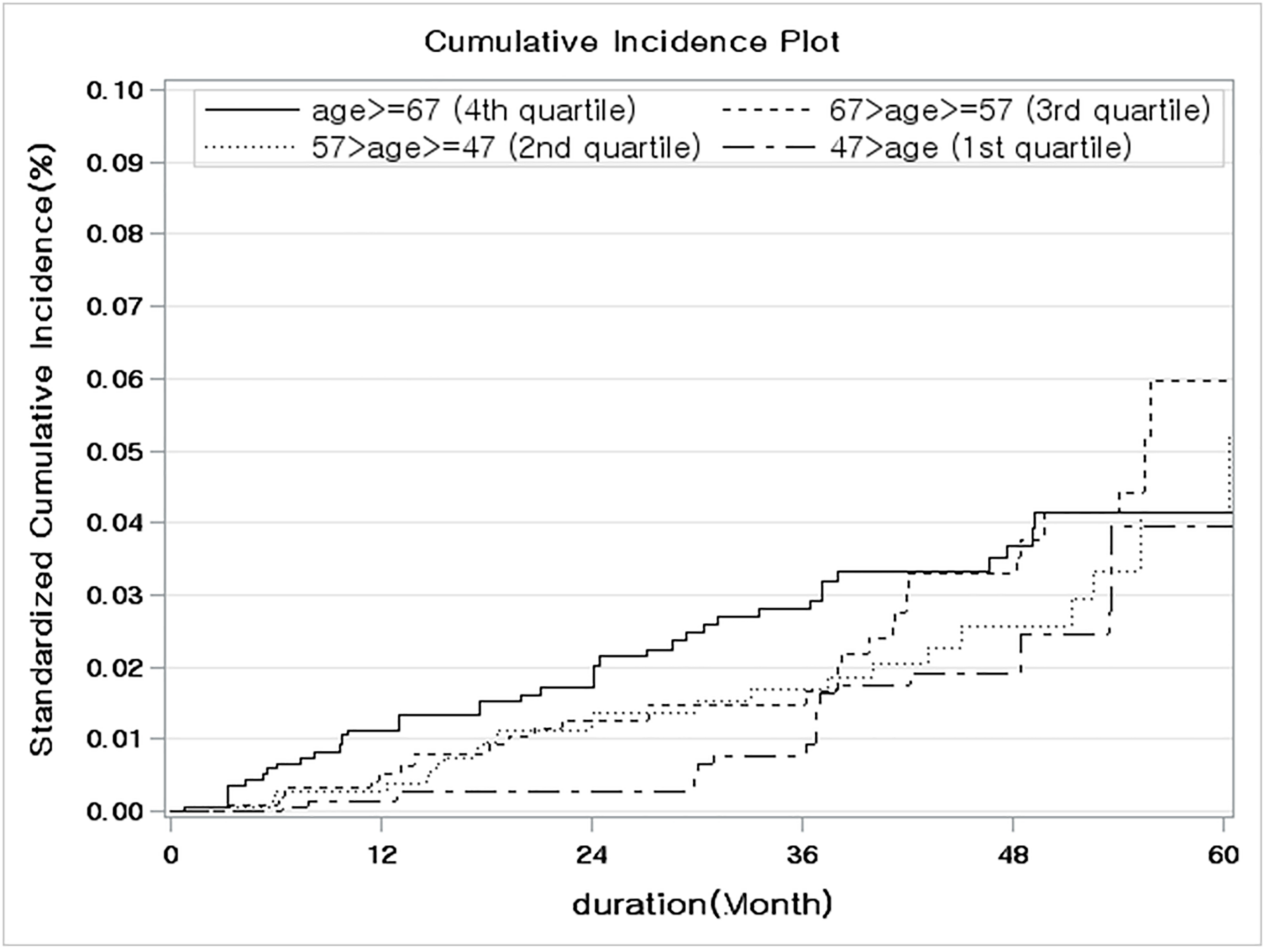
Comparison of the standardized cumulative incidence ratio controlling for the competing risk of death from any other cause stratified by age at the initiation of dialysis.

In order to clarify the risk for cancer among patients with ESRD, the crude cumulative incidence rate was calculated using the propensity score after adjustment to the inverse probability of treatment weight, considering ESRD-associated death as a competing risk. The difference between the crude and the standardized cumulative incidence rate at 5 years, stratified by baseline characteristics, is presented in Table 6. The 5-year crude and standardized cumulative incidence estimates of cancer were the highest among patients with a non-diabetic cause of ESRD, with a rate of 8.35% among patients with glomerulonephritis and 5.73% among patients with hypertension (Fig 1). Overall, the standardized cumulative incidence rate was higher among non-diabetic patients than among those with diabetes (5.41% *versus* 3.61%, respectively; S1 Fig), and among females, than among males (5.02% *versus* 3.66%, respectively, Fig 2). Although the highest standardized cumulative incidence rate of 5.15% was identified in the 3^rd^ quartile age group (Table 6), no significant effect of age (Fig 3) or dialysis modality (S2 Fig) on the SIR was identified.

**Table 6:** The five-year standardized cumulative incidence ratio controlled for the competing risk for death of any type of cancer.

		Crude	Standardized
Age at dialysis initiation (years)			
	≥67	4.61% (3.19–6.67)	4.16% (2.89–5.98)
	67 > ≥57	5.28% (3.34–8.35)	5.15% (3.34–7.94)
	57 > ≥47	4.16% (2.42–7.17)	4.15% (2.38–7.24)
	≥47	3.56% (2.03–6.25)	3.94% (2.22–7.01)
Sex			
	Male	3.73% (2.48–5.61)	3.66% (2.41–5.55)
	Female	5.05% (3.74–6.81)	5.02% (3.72–6.79)
Primary cause of ESRD			
	Diabetes	3.24% (2.21–4.74)	2.90% (1.94–4.33)
	Hypertension	5.73% (3.23–10.15)	5.73% (3.14–10.43)
	Glomerulonephritis	7.57% (4.52–12.66)	8.35% (5.07–13.75)
	Cystic kidney disease	2.41% (0.35–16.65)	1.57% (0.12–20.74)
	Unknown	1.17% (0.17–8.23)	1.00% (0.14–7.14)
	Others	4.75% (2.99–7.57)	4.99% (3.16–7.85)
Dialysis modality			
	Hemodialysis	4.24% (3.13,5.73)	4.46% (3.41–5.83)
	Peritoneal dialysis	4.94% (3.28,7.44)	4.63% (3.05–7.03)
Diabetes			
	DM (+)	3.37% (2.40–4.74)	3.61% (2.65–4.93)
	DM (-)	5.70% (4.07,8.00)	5.41% (3.89–7.54)

## Discussion

We conducted this large-prospective cohort study of patients with ESRD to provide an accurate incidence rate of cancer among this clinical population in Korea, to compare this incidence rate with the rate in the general population of Korea, and to establish criteria for cancer screening among this clinical population in Korea by calculating both crude and standardized cumulative incidence rates by using a competing risk analysis [13,19].

Our findings confirm previous reports of a specifically higher risk of urinary tract cancer, including renal cell carcinoma and bladder carcinoma, among patients with ESRD receiving hemodialysis or peritoneal dialysis than among the general population [3–8,14,24]. The selection of the control group for comparison between studies, in combination with region and ethnic effects, likely influenced differences in the magnitudes of risk hazard ratios for cancer in patient with ESRD between studies [3–7,14,24]. As an example, while a study involving American, European, Australian, and New Zealand patients reported a 1.5-fold increase in the rate of bladder carcinoma and 3.6-fold increase in renal cell carcinoma among patients with ESRD, compared to the general population [4], a study from Taiwan estimated these risk ratios at a 40.3-fold higher risk for kidney cancer [3]. Another retrospective Korean study identified 21 cases of urinary tract malignant tumors among 4,562 patients with ESRD, with 12 of these cases diagnosed with renal cell carcinoma [14]. A Japanese study of 13,497 patients on dialysis reported renal cell carcinoma as the most prevalent urinary malignancy, with an incidence rate of 0.61% [25]. In our study group, we identified a 4.7-fold higher risk for urinary tract cancer incidence, mainly attributable to kidney cancer among patients with ESRD than among the general population.

Although the biological factors contributing to the incidence of renal cell carcinoma among patients on long-term dialysis are debated, acquired cystic kidney disease is likely to be a main contributing factor [25–31]. Acquired cystic kidney disease is a relatively common complication of ESRD, with its likelihood increasing as a function of the severity of renal failure and longer duration of dialysis [28–31], the incidence rate increasing from 40% among patients with a dialysis history <3 years to 75% with 3 to 10 years of dialysis and 90% with >10 years [30,31]. In our study, the average monitoring period before detection of urinary tract cancer was 52 months, which is similar to the most frequently occurring gastrointestinal cancer detected, on average, at 58 months (Table 2). Therefore, screening for kidney and urinary system cancer should feasibly be performed within the first 5 years of dialysis, although further research is required to confirm an optimal screening method and interval for Korean patients with ESRD.

The prevalence of gastrointestinal cancer in our study was comparable to previously reported prevalence rates among Asian patients with ESRD in Japan [25] and Taiwan [3,6–8], as well as with epidemiological evidence of the highest worldwide prevalence rate of digestive system cancers being among East-Asian [32]. A clear estimate of the prevalence rate of digestive system cancers in East-Asian populations remains somewhat controversial, with the most recent study from Taiwan reporting a hazard ratio for colorectal cancer of 2.34, compared to a general population (p < 0.001) [3]. In the United States, colorectal cancer is the second most common cancer, based on a 5-year cumulative incidence by site-specific cancer, with a recent increase to a SIR of 1.27 [24]. Although colorectal cancer had the highest incidence rate in our study (16/116 cases; Table 2), the SIR for digestive system cancers in our study was only 0.607. Therefore, screening for colorectal cancer using endoscopy should be cautiously considered among patients with ESRD in Korea, considering the elevated risk for complications associated with the procedure itself and the bowel cleansing preparation in this clinical population, including hypovolemia and an associated hypotension [33,34]. These complications could accelerate thrombosis in the arteriovenous fistula used for dialysis, a condition that would be exacerbated by the hypovolemia during subsequent dialysis [34]. The large amount of cleansing agent administered could also result in fluid overload in patients with anuria.

Unlike previous studies [4–8], we accounted for the competing risk of ESRD-related death in our estimate of cumulative cancer incidence, this risk being significant in this population [3,13,24]. Furthermore, to minimize the biasing effects of different baseline characteristics among patients with ESRD, we estimated the propensity score for cancer incidence using logistic regression analysis, which controlled for age at the initiation of dialysis, sex, primary cause of ESRD, dialysis duration, dialysis modality, and diabetes. In reporting their 5-year cumulative incidence rate of any cancer of 9.48% in the United States, Butler et al. identified older age, male sex, and non-diabetic chronic kidney disease as risk factors, with the highest standardized cumulative incidence of cancer of 12.01% being among patients with ESRD due to glomerulonephritis, with a standardized cumulative incidence rate of 12.01% [24], which is similar to our rate of 8.35%. The specific higher risk for cancer among patients with non-diabetic chronic kidney disease has been previously reported and could reflect the higher risk of diabetic patients of dying from cardiovascular disease [1,2]. It is important to consider that the use of immunosuppressant therapy for glomerulonephritis may itself be a risk factor for cancer [35], with the risk of cancer increasing with the cumulative dose of immunosuppressant therapy [36–38]. Studies are warranted to specifically evaluate the biological associations between ESRD-related malignancies and long-term use of immunosuppressant therapy for glomerulonephritis prior to dialysis.

We identified a higher standardized cumulative risk of cancer among female than among male patients with ESRD. Reported sex-specific effects on cancer risk have been heterogeneous [3,6–8,24], with two recent studies accounting for competing risk for death reporting conflicting results [3,24]. Evidence of a higher overall risk for a worse clinical outcome and death from all causes for male patients with ESRD, compared to that for female patients [1,2,39], underlines the importance of using a competing risk analysis to accurately determine risk for cancer. Consistent with our findings, cohort studies in Taiwan reported a higher risk of cancer among female than among male patients with ESRD [3,6–8]. A possible specific association between being female and the use of Chinese herbal medicines has been suggested and warrants further investigation [40,41].

The limitations of our study were as follows. Foremost, the monitoring period was relatively short, which could explain the low incidence rate of cancer among our study group. Additionally, our sample size was small, with only 116 cases of cancer identified. It is likely that other cases of cancer were unreported. Therefore, future prospective studies with a larger sample size are needed to confirm our findings and the criteria for cancer screening among patients with ESRD in Korea. Currently, the screening guidelines for the general population defined by the Korea National Cancer Center (2015) do not include patients on dialysis [42]. Based on our results, we propose that an adequate cancer screening program for genitourinary cancer should be developed specifically for patients with ESRD in Korea, with consideration of the cost-effectiveness of such a program [43]. Screening programs should target women and patients with non-diabetic chronic kidney disease. Once screening programs are in place, the risk of cancer incidence among this clinical population can be more accurately evaluated.

## Supporting information

S1 Fig Comparison of the standardized cumulative incidence ratio considering competing risk according to the presence of diabetes. (TIF)

S2 Fig Comparison of the standardized cumulative incidence ratio controlling for the competing risk of death from any other cause stratified by dialysis modality. (TIF)

S1 Table The weight size of selected attribute for calculating standardized cumulative incidence using inverse probability of treatment weights (IPTW). (DOCX)

S2 Table Detailed information of newly developed cancer in patients with ESRD. (DOCX)

S1 File Supporting information_OVERALL DATA(N = 5235). (XLS)
