# Supplementary figures and images for: Cancer in Korean patients with end-stage renal disease: A 7-year follow-up
Source: PLoS One. 2017 Jul 10;12(7):e0178649. doi: 10.1371/journal.pone.0178649 (PMC5503228; doi:10.1371/journal.pone.0178649)

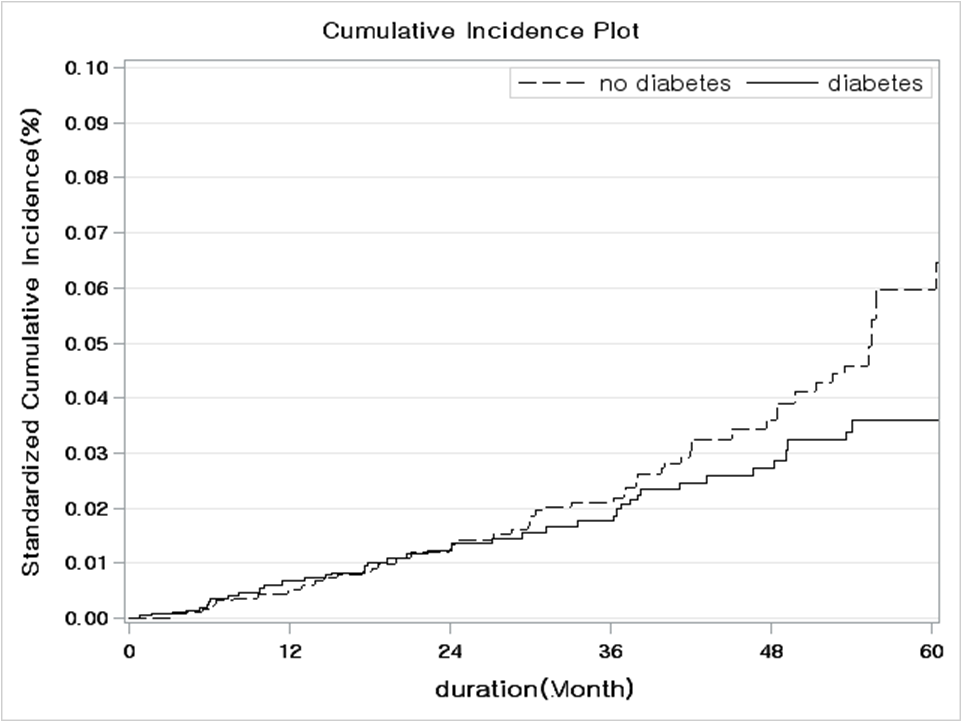

Supplement: S1 Fig — (TIF) [file pone.0178649.s001.tif]

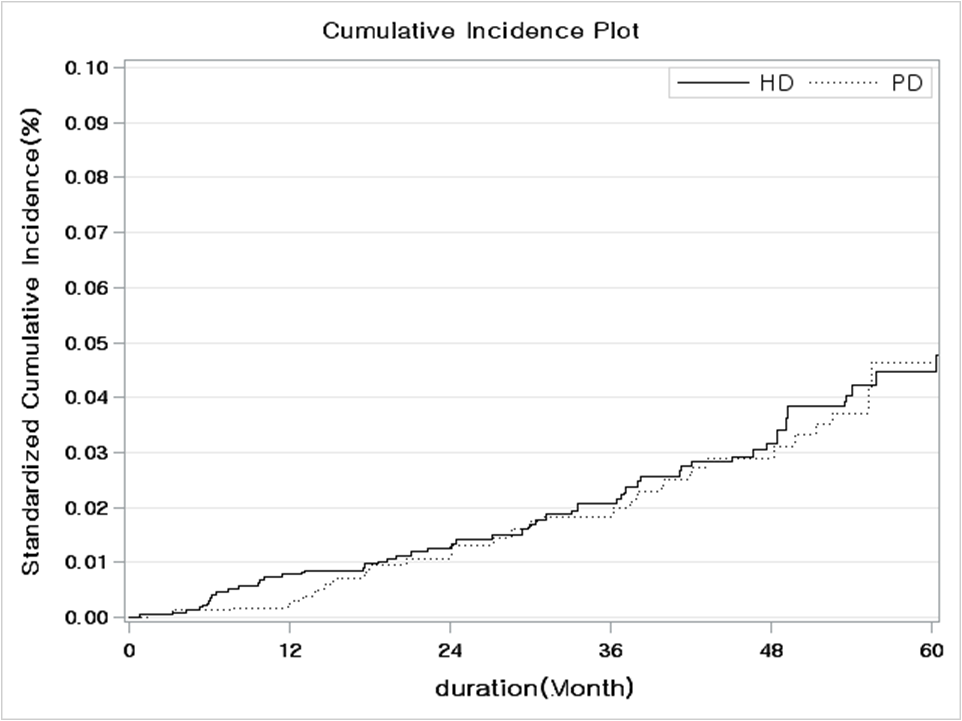

Supplement: S2 Fig — (TIF) [file pone.0178649.s002.tif]
