# Supplementary material for: Cancer in Korean patients with end-stage renal disease: A 7-year follow-up
Source: PLoS One. 2017 Jul 10;12(7):e0178649. doi: 10.1371/journal.pone.0178649 (PMC5503228; doi:10.1371/journal.pone.0178649)
Supplement: S1 Table — (DOCX) [file pone.0178649.s003.docx]

**S1 Table**. The weight size of selected attribute for calculating standardized cumulative incidence using inverse probability of treatment weights (IPTW)

|  | N | IPTW | Standard deviation | Median value |  | |
| --- | --- | --- | --- | --- | --- | --- |
|  |  |  |  |  | Minimal value | Maximal value |
| Age at dialysis initiation (years) | 4579 | 1.0142 | 0.1210 | 1.0005 | 0.8139 | 1.2281 |
| Sex | 4579 | 1.0015 | 0.3841 | 0.9194 | 0.4239 | 3.6044 |
| Primary cause of ESRD | 4579 | 0.9998 | 0.3405 | 0.9443 | 0.4473 | 2.6680 |
| Dialysis modality | 4579 | 1.1191 | 0.3982 | 1.0423 | 0.5997 | 2.9300 |
| Dialysis duration | 4579 | 1.0348 | 0.2015 | 0.9867 | 0.7084 | 1.8961 |
| Diabetes | 4579 | 1.0734 | 0.2781 | 1.0552 | 0.6304 | 1.5960 |
