# Supplementary material for: Cancer in Korean patients with end-stage renal disease: A 7-year follow-up
Source: PLoS One. 2017 Jul 10;12(7):e0178649. doi: 10.1371/journal.pone.0178649 (PMC5503228; doi:10.1371/journal.pone.0178649)
Supplement: S2 Table — (DOCX) [file pone.0178649.s004.docx]

**S2 Table. Detail information of newly developed cancer in patients with ESRD**

|  | Cancer location | N  (N= 116) | Cancer Stage at diagnosis (%) | | | | | | Treatment Type of Cancer | |  |
| --- | --- | --- | --- | --- | --- | --- | --- | --- | --- | --- | --- |
| Digestive | Stomach | 13 (11.2) | Unknown | | | | | 3 (23.1) | None | 1 (7.7) |  |
|  |  |  |  |  |  |  |  |  | Unknown | 1 (7.7) |  |
|  |  |  | In situ | | | | | 4 (30.8) | OP only | 8 (61.5) |  |
|  |  |  |  |  |  |  |  |  | RT or CT only | 2 (15.4) |  |
|  |  |  | Metastasis | | | | | 6 (46.2) | OP + RT or CT | 0 (0.0) |  |
|  |  |  |  |  |  |  |  |  | Others | 1 (7.7) |  |
|  | Colorectal | 16 (13.8) | Unknown | | | | 4 (25.0) | | None | 2 (12.5) | |
|  |  |  |  |  |  |  |  |  | Unknown | 2 (12.5) | |
|  |  |  | In situ | | | | 7 (43.8) | | OP only | 7 (43.8) | |
|  |  |  |  |  |  |  |  |  | RT or CT only | 1 (6.2) | |
|  |  |  | Metastasis | | | | 5 (31.2) | | OP + RT or CT | 0 (0.0) | |
|  |  |  |  |  |  |  |  |  | Others | 4 (25.0) | |
|  | Liver | 6 (5.2) | Unknown | | | 2 (33.3) | | | None | 1 (16.7) | |
|  |  |  |  |  |  |  |  |  | Unknown | 0 (0.0) | |
|  |  |  | In situ | | | 3 (50.0) | | | OP only | 0 (0.0) | |
|  |  |  |  |  |  |  |  |  | RT or CT only | 2 (33.3) | |
|  |  |  | Metastasis | | | 1 (16.7) | | | OP + RT or CT | 0 (0.0) | |
|  |  |  |  |  |  |  |  |  | Others | 3 (50.0) | |
|  | Pancreatobiliary | 2 (1.7) | Unknown | | 1 (50.0) | | | | None | 0 (0.0) | |
|  |  |  |  |  |  |  |  |  | Unknown | 1 (50.0) | |
|  |  |  | In situ | | 0 (0.0) | | | | OP only | 0 (0.0) | |
|  |  |  |  |  |  |  |  |  | RT or CT only | 1 (50.0) | |
|  |  |  | Metastasis | | 1 (50.0) | | | | OP + RT or CT | 0 (0.0) | |
|  |  |  |  |  |  |  |  |  | Others | 0 (0.0) | |
|  | Other GI tract | 2 (1.7) | Unknown | 0 (0.0) | | | | | None | 1 (50.0) | |
|  |  |  |  |  |  |  |  |  | Unknown | 0 (0.0) | |
|  |  |  | In situ | 2 (100.0) | | | | | OP only | 1 (50.0) | |
|  |  |  |  |  |  |  |  |  | RT or CT only | 0 (0.0) | |
|  |  |  | Metastasis | 0 (0.0) | | | | | OP + RT or CT | 0 (0.0) | |
|  |  |  |  |  |  |  |  |  | Others | 0 (0.0) | |
| Respiratory | Lung | 14 (12.1) | Unknown | 2 (14.3) | | | | | None | 3 (21.4) | |
|  |  |  |  |  |  |  |  |  | Unknown | 2 (14.3) | |
|  |  |  | In situ | 5 (35.7) | | | | | OP only | 2 (14.3) | |
|  |  |  |  |  |  |  |  |  | RT or CT only | 5 (35.7) | |
|  |  |  | Metastasis | 7 (50.0) | | | | | OP + RT or CT | 0 (0.0) | |
|  |  |  |  |  |  |  |  |  | Others | 2 (14.3) | |
|  | Larynx | 1 (0.9) | Unknown | 0 (0.0) | | | | | None | 0 (0.0) | |
|  |  |  |  |  |  |  |  |  | Unknown | 0 (0.0) | |
|  |  |  | In situ | 0 (0.0) | | | | | OP only | 0 (0.0) | |
|  |  |  |  |  |  |  |  |  | RT or CT only | 1 (100.0) | |
|  |  |  | Metastasis | 1 (100.0) | | | | | OP + RT or CT | 0 (0.0) | |
|  |  |  |  |  |  |  |  |  | Others | 0 (0.0) | |
| Reproductive | Uterine | 2 (1.7) | Unknown | 0 (0.0) | | | | | None | 0 (0.0) | |
|  |  |  |  |  |  |  |  |  | Unknown | 0 (0.0) | |
|  |  |  | In situ | 2 (100.0) | | | | | OP only | 1 (50.0) | |
|  |  |  |  |  |  |  |  |  | RT or CT only | 0 (0.0) | |
|  |  |  | Metastasis | 0 (0.0) | | | | | OP + RT or CT | 0 (0.0) | |
|  |  |  |  |  |  |  |  |  | Others | 1 (50.0) | |
|  | Prostate | 6 (5.2) | Unknown | 1 (16.7) | | | | | None | 0 (0.0) | |
|  |  |  |  |  |  |  |  |  | Unknown | 1 (16.7) | |
|  |  |  | In situ | 1 (16.7) | | | | | OP only | 4 (66.7) | |
|  |  |  |  |  |  |  |  |  | RT or CT only | 1 (16.7) | |
|  |  |  | Metastasis | 4 (66.7) | | | | | OP + RT or CT | 0 (0.0) | |
|  |  |  |  |  |  |  |  |  | Others | 0 (0.0) | |
|  | Others | 1 (0.9) | Unknown | 0 (0.0) | | | | | None | 0 (0.0) | |
|  |  |  |  |  |  |  |  |  | Unknown | 0 (0.0) | |
|  |  |  | In situ | 0 (0.0) | | | | | OP only | 1 (100.0) | |
|  |  |  |  |  |  |  |  |  | RT or CT only | 0 (0.0) | |
|  |  |  | Metastasis | 1 (100.0) | | | | | OP + RT or CT | 0 (0.0) | |
|  |  |  |  |  |  |  |  |  | Others | 0 (0.0) | |
| Endocrine | Thyroid | 11 (9.5) | Unknown | 1 (9.1) | | | | | None | 0 (0.0) | |
|  |  |  |  |  |  |  |  |  | Unknown | 1 (9.1) | |
|  |  |  | In situ | 4 (36.4) | | | | | OP only | 9 (81.8) | |
|  |  |  |  |  |  |  |  |  | RT or CT only | 0 (0.0) | |
|  |  |  | Metastasis | 6 (54.5) | | | | | OP + RT or CT | 1 (9.1) | |
|  |  |  |  |  |  |  |  |  | Others | 0 (0.0) | |
|  | Others | 1 (0.9) | Unknown | 0 (0.0) | | | | | None | 0 (0.0) | |
|  |  |  |  |  |  |  |  |  | Unknown | 0 (0.0) | |
|  |  |  | In situ | 1 (100.0) | | | | | OP only | 1 (100.0) | |
|  |  |  |  |  |  |  |  |  | RT or CT only | 0 (0.0) | |
|  |  |  | Metastasis | 0 (0.0) | | | | | OP + RT or CT | 0 (0.0) | |
|  |  |  |  |  |  |  |  |  | Others | 0 (0.0) | |
| Other organ | Breast | 9 (7.8) | Unknown | 1 (11.1) | | | | | None | 0 (0.0) | |
|  |  |  |  |  |  |  |  |  | Unknown | 1 (11.1) | |
|  |  |  | In situ | 5 (55.6) | | | | | OP only | 5 (55.6) | |
|  |  |  |  |  |  |  |  |  | RT or CT only | 0 (0.0) | |
|  |  |  | Metastasis | 3 (33.3) | | | | | OP + RT or CT | 3 (33.3) | |
|  |  |  |  |  |  |  |  |  | Others | 0 (0.0) | |
|  | Skin | 3 (2.6) | Unknown | 1 (33.3) | | | | | None | 2 (66.7) | |
|  |  |  |  |  |  |  |  |  | Unknown | 0 (0.0) | |
|  |  |  | In situ | 2 (66.7) | | | | | OP only | 1 (33.3) | |
|  |  |  |  |  |  |  |  |  | RT or CT only | 0 (0.0) | |
|  |  |  | Metastasis | 0 (0.0) | | | | | OP + RT or CT | 0 (0.0) | |
|  |  |  |  |  | | | | | Others | 0 (0.0) | |

ESRD, end-stage renal disease; GI tract, gastrointestinal tract; OP, operation, RT, radiotherapy, CT, chemotherapy
